# Lymphatic Vessel Regression and Its Therapeutic Applications: Learning From Principles of Blood Vessel Regression

**DOI:** 10.3389/fphys.2022.846936

**Published:** 2022-03-22

**Authors:** Faisal Masood, Rohan Bhattaram, Mark I. Rosenblatt, Andrius Kazlauskas, Jin-Hong Chang, Dimitri T. Azar

**Affiliations:** Department of Ophthalmology and Visual Sciences, Illinois Eye and Ear Infirmary, College of Medicine, University of Illinois at Chicago, Chicago, IL, United States

**Keywords:** lymphatic vessel regression, blood vessel regression, antilymphangiogenesis, lymphangiogenesis, angiogenesis

## Abstract

Aberrant lymphatic system function has been increasingly implicated in pathologies such as lymphedema, organ transplant rejection, cardiovascular disease, obesity, and neurodegenerative diseases including Alzheimer’s disease and Parkinson’s disease. While some pathologies are exacerbated by lymphatic vessel regression and dysfunction, induced lymphatic regression could be therapeutically beneficial in others. Despite its importance, our understanding of lymphatic vessel regression is far behind that of blood vessel regression. Herein, we review the current understanding of blood vessel regression to identify several hallmarks of this phenomenon that can be extended to further our understanding of lymphatic vessel regression. We also summarize current research on lymphatic vessel regression and an array of research tools and models that can be utilized to advance this field. Additionally, we discuss the roles of lymphatic vessel regression and dysfunction in select pathologies, highlighting how an improved understanding of lymphatic vessel regression may yield therapeutic insights for these disease states.

## Introduction

Increasingly implicated in various pathologies, the lymphatic system plays important roles in immune cell transport, gastrointestinal nutrient absorption, and interstitial fluid transport (Alitalo et al., 2005; Tammela and Alitalo, 2010; Petrova and Koh, 2020; Landau et al., 2021). The identification of lymphatic-specific markers such as vascular endothelial growth factor receptor 3 (VEGFR-3), lymphatic vessel endothelial hyaluronan receptor 1 (LYVE-1), prospero-related homeobox 1 (Prox-1), and podoplanin in the late 1990s was instrumental in paving the way for research on lymphangiogenesis, or the production of new lymphatic vessels (LVs) from extant LVs. There has been a steady stream of research conducted on lymphangiogenesis since the identification of lymphatic endothelial cell (LEC) markers, culminating in approximately 4152 publications by 2021; however, despite its importance in phenomena such as tumor cell metastasis (Detmar and Hirakawa, 2002; Stacker et al., 2002, 2014) and organ transplantation rejection (Yamakawa et al., 2018), the field of lymphangiogenesis is largely overshadowed by its blood vessel (BV) counterpart, angiogenesis. In 2021, approximately 124,224 publications exist in the field of angiogenesis, nearly 30-fold more publications than for lymphangiogenesis.

This exploration of vessel formation and progression is only one side of the coin; a detailed understanding of the regression of both newly formed and extant vasculature is the other half of this story. At the time of writing, there are approximately 525 publications on LV regression compared to 25,037 publications on BV regression, a 47.8-fold difference. The phenomenon of BV regression and its therapeutic implications were explored as early as the 1940s and 50s, roughly a half century prior to the discovery of the aforementioned markers of LECs that enabled the study of LV regression (Terry, 1942; Michaelson and Schreiber, 1956), with researchers subsequently proposing that modulation of vascular endothelial growth factor (VEGF) signaling pathways and other angiogenic pathways would prove fruitful in anti-tumor therapies (Kowanetz and Ferrara, 2006; Kong et al., 2017).

Considering the discovery of increased LV density in certain pathologies, such as inflammatory bowel disease (Zhang et al., 2021), where LV regression could be therapeutic, in addition to the identification of decreased lymphatic clearance in other pathologies, such as Alzheimer’s disease (Tarasoff-Conway et al., 2015; Petrova and Koh, 2020), a clear understanding of the mechanisms underlying LV regression is urgently needed. Herein, we summarize what is known about LV regression, extend insights from the prolific field of BV regression to shed light on LV regression, discuss the implications of the newly identified roles of lymphatics in a variety of pathologies, and describe research models and modalities that can be utilized to further advance the understudied field of LV regression. By applying salient BV regression principles, we explore how concepts such as homeostasis, inflammation, negative feedback, and apoptosis may elucidate findings in LV regression.

### (Lymph)angiogenesis in Physiology and Disease

Angiogenesis, the growth and development of new BVs from extant BVs, is a well-studied and characterized process (Carmeliet and Jain, 2011). While vasculogenesis refers to the development of the heart and basic vascular structures (Patan, 2004), angiogenesis is the process by which this network expands and matures into a fully functional system. The primary driver of angiogenesis is VEGF-A, which binds to VEGF receptors 1 and 2 (VEGFR-1/2) and induces vasodilation and cellular permeability. A detailed overview of the protein families and molecular mediators implicated in angiogenesis can be found in the review by Carmeliet and Jain (2011). Lymphangiogenic progression follows growth patterns like those observed in angiogenesis, with some key distinctions (Adams and Alitalo, 2007; Lohela et al., 2009). Similar to VEGF-A being the primary factor behind angiogenesis, VEGF-C is the driving factor for lymphangiogenesis, with VEGF-D exhibiting a lesser effect. A detailed mechanism of lymphangiogenesis is expertly reviewed by Oliver et al. (2020).

The lymphatic system plays an important role in both the vascular and immune systems. It regulates and returns fluid to the venous system while also transporting immune cells to appropriate locations. Lymph nodes are key in antigen presentation and generating humoral and cell-mediated immune responses. Although necessary for healthy tissue homeostasis, the development of lymphatic vasculature can also contribute to immunopathologies, such as chronic autoimmune disease (Kesler et al., 2013). In such chronic inflammatory states, lymphangiogenesis can create tertiary lymphatic organs (TLOs), which are very similar to normal lymph nodes. TLOs have been found in organ transplant recipients and corneal transplant recipients as well as in patients with rheumatoid arthritis and inflammatory bowel disease (Kesler et al., 2013). Pathologic lymphatic drainage is also a mechanism for tumor metastasis (Kesler et al., 2013). Tumor cells can induce lymphangiogenesis to connect to existing lymphatic vasculature, thereby increasing the risk of metastasis (Karpanen et al., 2001; Skobe et al., 2001). Although tumor-induced lymphangiogenesis may result in dysfunctional intratumoral lymphatics, Padera et al. (2002) demonstrated that VEGF-C–induced lymphangiogenesis promotes metastasis through the functional lymphatics formed at the tumor border. Unlike treatments targeting BV-mediated metastasis, anti-lymphangiogenic therapy is still underdeveloped clinically (Kesler et al., 2013; Yamakawa et al., 2018).

### Blood and Lymphatic Vessel Regression

**FIGURE 1:**
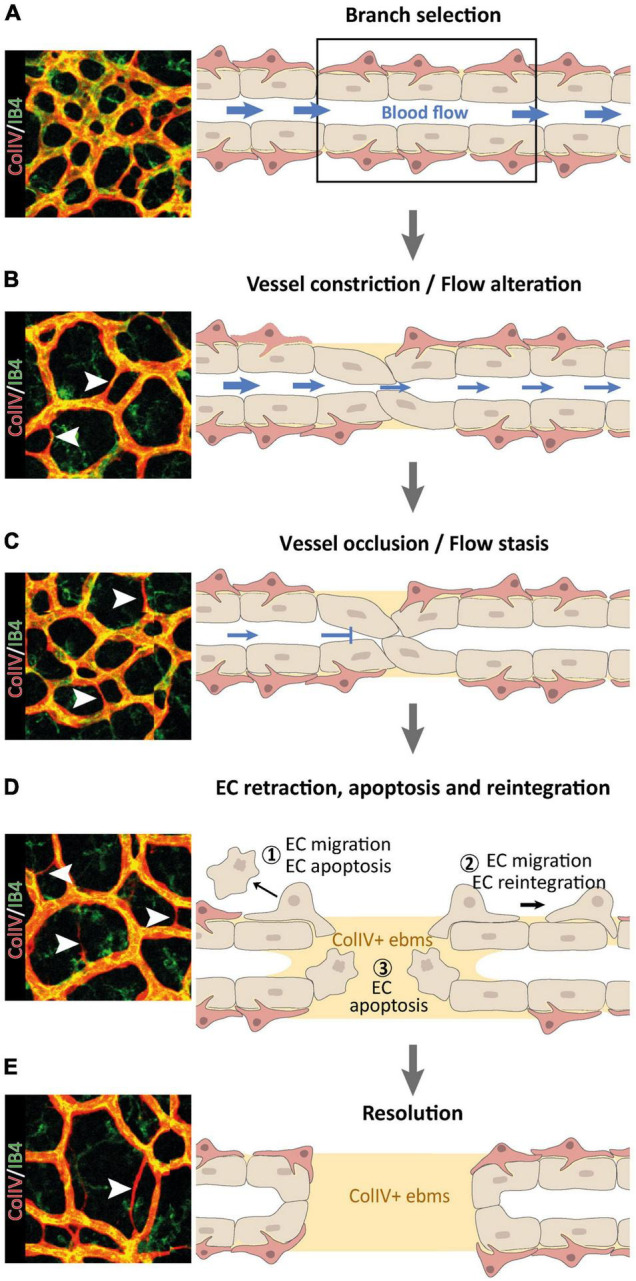
Multi-step nature of vessel pruning and regression processes, reproduced from Korn and Augustin (2015). **(A–D)** Following the primarily blood flow-driven selection of a BV branch for regression **(A)**, the BV constricts **(B)** until it occludes **(C)** and the blood flow ceases **(D)**. ECs within regressing vessel segments may retract and undergo apoptosis (1), or they may migrate away to re-integrate elsewhere (2), leaving behind collagen V (ColIV) + empty basement membrane sleeves (ebms). Retracting ECs can disintegrate from the vascular network and undergo EC apoptosis due to detachment from the basement membrane (3). **(E)** In a final resolution step, regression of the selected BV branch is completed, leaving a remodeled vascular network behind.

Blood vessel regression is a complex, multifaceted process that is still poorly understood compared to angiogenic growth (Korn and Augustin, 2015). BV regression has been studied in the context of vessel pruning, which is the process of microvessel regression during BV maturation and is known to involve a diverse set of molecular markers and pathways. Korn and Augustin described multiple mechanisms of BV regression (Figure 1), with VEGF, Wnt/Notch, angiopoietin (Ang)/tyrosine kinase with immunoglobulin-like and EGF-like domains (TIE), and other signaling pathways playing key roles (Korn and Augustin, 2015). Claxton and Fruttiger (2003) used an induced hyperoxia mouse model in which VEGF was downregulated to demonstrate that FGF2, ANG2, platelet-derived growth factor (PDGF), and Delta-like canonical Notch ligand 4 (DLL4) are involved in maintaining vessel stability independent of VEGF. A mouse model study of Parkinson’s disease focused specifically on the vascular pathologic effect, and VEGF was shown to maintain mature vessels and induce plasticity in response to external factors (Elabi et al., 2021). Korn et al. (2014) demonstrated how the Wnt/Notch signaling pathway also has a key role in BV maturation and stabilization (Korn and Augustin, 2015), with improper signaling potentially resulting in inducing spontaneous vessel regression and death. DLL4 expressed by ECs can bind with Notch as well and downregulate VEGF, a key signaling event for capillary remodeling (Lobov et al., 2011). As Notch ligand interactions are primarily involved in cell death and apoptosis (Miele and Osborne, 1999), there appear to be multiple approaches to induce cell apoptosis. These pathways occur concurrently with the Ang/TIE2 pathway, which regulates vessel plasticity, and are crucial for vessel pruning and ending the vessel maturation process (Welti et al., 2013). Other pathways include Hairy/enhancer-of-split related with YRPW motif protein (HEY)/p53, cysteine-rich transmembrane BMP regulator 1 (CRIM1), and C-X-C motif chemokine ligand 10 (CXCL10)/C-X-C motif chemokine receptor 3 (CXCR3), which increase VEGFR-1, stimulate VEGF-A signaling, and detach ECs to prepare for apoptosis, respectively, all of which are key processes in BV pruning and regression (Bodnar et al., 2009; Fan et al., 2014). Researchers have observed a unique pattern in these complex pathways, termed the “anti-angiogenic switch,” which represents a modified form of the “angiogenic switch” concept from tumor vasculature. Essentially, the mere addition or inhibition of VEGF does not necessarily change the angiogenic direction. Gosain et al. (2006) assessed the abilities of exogenous VEGF, FGF, PDGF, and a combination of VEGF and FGF to reverse vascular regression. They only observed a transient increase in vessel density, and regression continued after an observed period. Conversely, Irani et al. (2017) tested the ability of an anti-VEGF-B antibody fragment to induce BV regression and found that the antibody fragment alone could not significantly reduce BV density. These findings indicate that signaling pathways are not simply “pro” or “anti” angiogenesis; instead, a myriad of concurrent signals with a basal level of VEGF make angiogenic regression a resilient state (Elabi et al., 2021).

Physiological BV regression occurs to facilitate the maturation of developing blood vessels and to prevent excessive BV growth (Korn and Augustin, 2015). There are many instances in which blood vessels regress almost completely in healthy states. Hyaloid vessels, which are present in the eye during embryonic development, completely regress after complete eye development, primarily through pathways involving Wnt signaling (Wang et al., 2019a,b). The menstrual cycle also presents a unique BV growth and regression cycle; during luteolysis of the ovarian corpus luteum, Goede et al. (1998) observed an association between upregulated Ang2 expression (relative to Ang1) and BV regression. Another unique physiological example of BV regression is in the context of embryological finger patterning, a process involving apoptosis of embryologic interdigital webbing to form free digits (Hurle et al., 1985). Prior to physiologic apoptosis of interdigital webbing, robust interdigital angiogenesis increases the local oxygen concentration, resulting in the generation of reactive oxygen species (ROS) that impart cellular damage (Eshkar-Oren et al., 2015; Cordeiro et al., 2019). This oxidative stress culminates in interdigital webbing apoptosis, which is followed by remodeling and regression of the interdigital vasculature. Pathological BV regression has been seen in a wide variety of conditions. Retinopathy of prematurity (Keshet, 2003), pulmonary fibrosis (Ebina et al., 2004; Parra et al., 2005; Johnson and DiPietro, 2013; Barratt and Millar, 2014), chronic kidney disease (Polichnowski, 2018), kidney microvessel rarefaction (Goligorsky, 2010; Chade, 2013), Parkinson’s disease (Elabi et al., 2021), spinal cord injury (Trivedi et al., 2016), and initial tumor BV co-option (Holash et al., 1999) have all been linked to BV regression during specific pathological time frames (Table 1).

**TABLE 1:** Pathologic blood vessel (BV) regression.

Organ system	References	pathologies/disease	Mechanism	Affected BVs
Eye	Keshet, 2003	Retinopathy of prematurity	Oxygen supplementation in premature infants can lead to hyperoxia-induced underexpression of VEGF, resulting in pathologic regression of retinal BVs. As the infant returns to room air, the deficit in retinal BVs results in a relatively hypoxic state, causing robust angiogenesis. This compensatory response results in the excessive formation of leaky BVs that may infiltrate the inner layer of the retina and vitreous, potentially causing retinal detachment and blindness.	Retinal BVs
Lung	Parra et al., 2005; Johnson and DiPietro, 2013; Barratt and Millar, 2014	Fibrosis	Injury to alveolar epithelium results in an inflammatory response and robust angiogenesis; ongoing inflammation results in fibrotic parenchymal remodeling and vascular regression by apoptosis of VECs.	Pulmonary microvessels
Kidney	Goligorsky, 2010; Fligny and Duffield, 2013; Polichnowski, 2018	CKD, Microvessel rarefaction	Altered peritubular capillary caliber, increased recruitment of renal immune cells, altered mechanical forces, and potentially other mechanisms contribute to pericyte dysfunction and detachment from BVs, ultimately leading to BV rarefaction and regression.	Tubular microvessels
Brain	Elabi et al., 2021	Parkinson’s, Spinal cord injury	Pericyte activation leads to compensatory angiogenesis, followed by regression. Immediate regression could be due to impaired Wnt/ß-catenin signaling, and lack of MMP-2 in spinal cord injury can lead to angiogenic regression (again possibly due to pericyte disruption).	Brain and spine BVs
Cancer	Holash et al., 1999	Initial tumor co-option and BV regression	Some subset of malignancies may initially co-opt into extant vasculature, resulting in BV regression, local hypoxia, and subsequent angiogenesis. BV regression was physically characterized by separation of ECs from supporting mural cells and molecularly characterized by the up-regulation of Ang-2 in the absence of VEGF. The investigators hypothesized that this upregulation of Ang-2 may serve as a host defense mechanism to mark the coopted BVs for regression	Tumor-coopted BVs

*VEC, vascular endothelial cell; CKD, chronic kidney disease; MMP-2, matrix metalloproteinase-2.*

The phenomenon of LV regression is not well understood compared to BV regression. The exact mechanisms responsible for LV regression are not well defined, but TH1- and TH2-derived cytokines have been shown to play a role in negative regulation of lymphangiogenesis (Shao and Liu, 2006; Kataru et al., 2011; Savetsky et al., 2015). LV regression has been observed in experimental settings. In a study by Zhang et al. (2011) lymphatic regression was found to be transient as vessels eventually grew back, but this is not true for all LV regression states. Shi et al. (2020) observed LV regression due to aqueous humor in both healthy and inflamed states. They linked alpha-melanocyte–stimulating hormone (a-MSH), vasoactive intestinal peptide (VIP), thrombospondin-1 (TSP-1), transforming growth factor beta (TGF-β), and Fas ligand (FasL) found in aqueous humor to LV regression, but the roles of these factors in induced LV regression have yet to be confirmed (Shi et al., 2020).

Similar to LV regression mechanism knowledge, the knowledge of LV functions in both physiology and pathology is limited. LV regression in the endometrium has been linked to the menstrual cycle (Tomita and Mah, 2014), as Girling and Rogers found fewer vessels in the functionalis layer and reduced vessel density in the subepithelium in a pig uterus model (Girling and Rogers, 2005). Tomita and Mah (2014) demonstrate that LVs in the functionalis layer undergo a cycle of “proliferation and degeneration” that is synchronized with cyclic menstrual changes of endometrial arteries. Additionally, it has been noted that embryologic pulmonary LVs undergo physiological regression, with a persistence of these vessels implicated in the pathophysiology behind pulmonary lymphangiectasia (Esther and Barker, 2004). As mentioned above, promotion of LV regression was also observed in relation to eye development and aqueous humor (Shi et al., 2020). Although limited, these physiological and pathological examples of LV regression can be probed and further dissected to potentially yield context-specific information on the mechanisms of LV regression.

## Learning From Blood Vessel Regression

As discussed in the previous Section “Pathologies Involving Lymphangiogenic Regression and Dysfunction,” much remains to be discovered regarding LV regression. Here, we identify salient BV regression principles, extend these concepts to LV regression, and highlight pertinent lymphatic literature that supports these claims (Table 2).

**TABLE 2:** Extending BV regression hallmarks to lymphatic vessels (LV) regression.

Overarching theme	References	Insights for LV regression
Survival signals and homeostasis	Bergers et al., 2003; Keshet, 2003; Lee et al., 2007; Murakami, 2012; Ji, 2014; Korn and Augustin, 2015; Zhong et al., 2017; Fallah and Rini, 2019; Schito, 2019; Iyer et al., 2020; Stritt et al., 2021	Although largely uncharacterized, it is established that pro-survival and homeostatic signaling occur in LVs. The impact of absent shear stress and interstitial fluid flow on LV regression deserves further exploration. In contrast to BVs’ dependence on autocrine VEGF-A for survival, only the intestinal and meningeal lymphatics require VEGF-C signaling for maintenance and survival.
Inflammation	Folkman et al., 1983; Cursiefen et al., 2006; Logie et al., 2010; Kelley et al., 2011; Steele et al., 2011; Martínez-Corral et al., 2012; Mumprecht et al., 2012; Liao and von der Weid, 2014; Sajib et al., 2018; Filiberti et al., 2020; Ocansey et al., 2021	Inflammation serves as a stimulus for both angiogenesis and lymphangiogenesis. Inflammatory cytokines such as IL-6, IL-8, and TNF-α, have been implicated in neovascularization. Suppressing inflammation with glucocorticoids can prevent neovascularization. Further characterization of the effects of glucocorticoids through continuous live-imaging may allow for elucidation of the link between anti-inflammatory treatments and LV regression.
Anti-angiogenic switch and negative feedback	Lingen et al., 1996; Alitalo and Carmeliet, 2002; Watanabe et al., 2004; Gosain et al., 2006; Sato and Sonoda, 2007; Kataru et al., 2011; Wietecha et al., 2011; Shirasuna et al., 2012	Just as in BVs, negative feedback mechanisms likely regulate the balance between a lymphangiogenic and anti-lymphangiogenic state. Thus far, paracrine T-cell signaling through interferon-γ and vasohibin-1 inhibition of VEGF-A induced lymphangiogenesis have been implicated as potential negative feedback modulators of lymphangiogenesis. Endogenous mediators of the anti-lymphangiogenic switch remain largely uncovered. Further characterization of these molecules may reveal potent drivers of LV regression.
Apoptosis of regressing vessels	Ito and Yoshioka, 1999; Zhu et al., 2000; Mäkinen et al., 2001; Kim et al., 2010; Franco et al., 2015; Korn and Augustin, 2015; Watson et al., 2017; Gur-Cohen et al., 2019; Schafer et al., 2020; Hou et al., 2021	In certain contexts, apoptosis is a primary driver of BV regression. In other cases, apoptosis is a result of another determinant of BV regression. In another subset of cases, migration of VECs into neighboring BVs allows for a cell death-independent mechanism of BV regression. Although studies on the role of apoptosis in LV regression are currently lacking, it is possible that these mechanisms of BV regression hold similarly true in LV regression. Further work is required to characterize the nuances of apoptosis in LV regression.

### Survival Signals and Homeostasis

**FIGURE 2:**
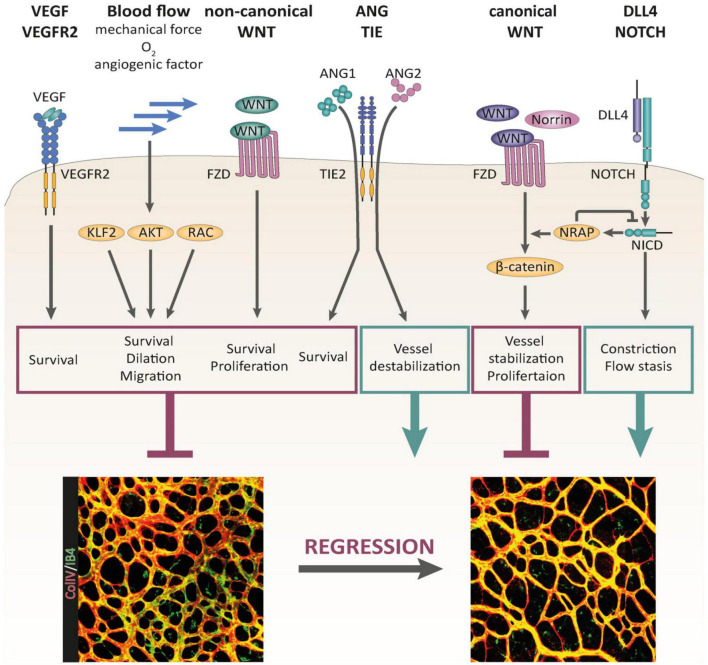
Signaling pathways controlling BV pruning and regression, reproduced from Korn and Augustin (2015), Korn and Augustin (2015). Multiple signaling pathways have been identified as regulators of BV regression. VEGF/VEGFR-2 signaling, non-canonical WNT signaling, and blood flow-induced signaling serve as critical maintenance factors of the vasculature that are involved in the control of BV regression. Canonical WNT signaling stabilizes the vascular network and promotes EC proliferation. DLL4/Notch signaling supports BV regression by promoting BV constriction and flow stasis. The outcome of ANG/TIE signaling during BV remodeling is context dependent. Whereas ANG1 supports EC survival, ANG2 destabilizes the vascular network, driving it into regression in the absence of survival factor activity (e.g., VEGF).

The basal state of BVs is one of quiescence and homeostasis. Stabilized networks are remarkably unperturbed by both pro- and anti-angiogenic factors various protective mechanisms ensure that a vascular network does not undergo inappropriate pruning or proliferation. These stabilizing factors include pericyte coverage (Bergers et al., 2003), vascular endothelial (VE)-cadherin cell–cell junctions (Murakami, 2012), and autocrine VEGF-A signaling (Lee et al., 2007). Factors promoting either homeostasis or BV regression are illustrated in Figure 2. For a more detailed summary of the factors involved in BV homeostasis and quiescence, see the review by Murakami (2012). The signaling required to maintain BV homeostasis is a result of upstream phenomena. For example, HIF-1 alpha accumulates in cells exposed to hypoxic conditions and consequently leads to the transcription of pro-angiogenic genes including VEGF-A (Fallah and Rini, 2019). Absence of this upstream requirement for homeostasis can lead to dramatic BV regression. For example, in retinopathy of prematurity, administration of high concentration oxygen to premature infants obliterates the local hypoxic signaling required to maintain the immature retinal vessels, leading to pathological BV regression (Keshet, 2003). Another upstream phenomenon that promotes BV survival signaling is shear stress imparted by laminar blood flow in a properly perfused vessel (Korn and Augustin, 2015).

**FIGURE 3:**
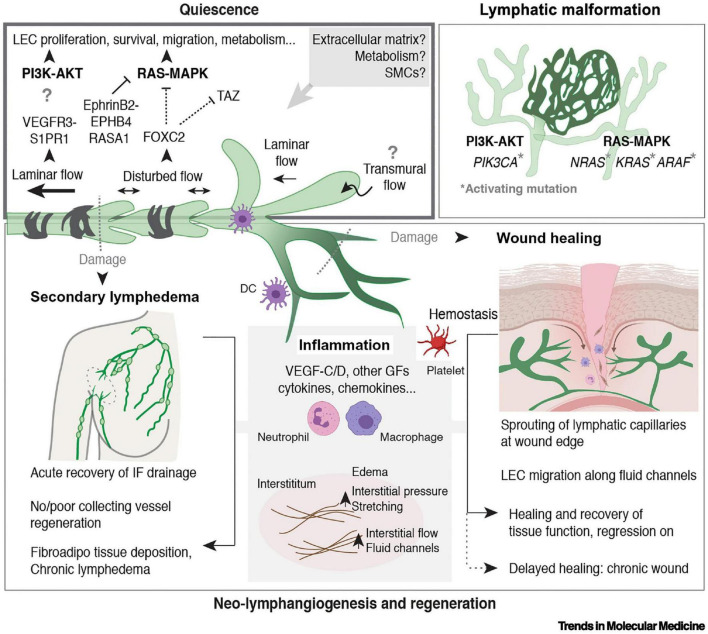
Regulation of LV quiescence and growth in adults, reproduced from Stritt et al. (2021). Mechanisms that regulate the maintenance of LEC quiescence (upper left box) and the reactivation of LV growth and regeneration in adult tissues are depicted. Somatic activating mutations in genes encoding components of the major mitogenic phosphoinositide 3-kinase (PI3K) and rat sarcoma viral oncogene (RAS)–mitogen-activated protein kinase (MAPK) pathways cause lymphatic malformations (upper right box). Inflammation is a major driver of neo-lymphangiogenesis (in gray in the lower box). Processes associated with wounding and damage to lymphatic capillaries or collecting LVs are indicated (lower box). Figure created with the help of BioRender.com. Abbreviations: AKT, protein kinase B; ARAF, A-Raf proto-oncogene serine/threonine kinase; DC, dendritic cell; EPHB4, ephrin receptor B4; FOXC2, Forkhead box C2; GF, growth factor; IF, interstitial fluid; KRAS, Kirsten RAS; NRAS, neuroblastoma RAS; RASA1, Ras p21 protein activator 1; S1PR1, sphingosine 1-phosphate receptor 1; SMC, smooth muscle cell; TAZ, Tafazzin phospholipid-lysophospholipid transacetylase; VEGFR-3, vascular endothelial growth factor receptor 3.

Extending these findings to LVs, it is certain that these vessels similarly experience pro-survival signaling, the disruption of which can lead to LV regression (Figure 3). Although the LV network experiences less shear stress than the BV network, Stritt et al. (2021) showed that LVs have greater sensitivity to shear stress and further elucidated the mechanisms by which shear stress may lead to LEC proliferation *in vitro*. In addition to LEC proliferation and potential survival, shear stress has also been implicated in the survival of lymphatic valves (Iyer et al., 2020). Although not as clear as in the BV context, shear stress signaling on a perfused LV has been seen to play a role in LV proliferation, and further studies may evaluate its role in LV regression. Additionally, similar to the angiogenic context, local hypoxia and subsequent HIF signaling induces lymphangiogenesis through increased expression of VEGF-C and VEGF-D (Ji, 2014; Schito, 2019). This commonality between angiogenesis and lymphangiogenesis may allude to normoxia as a survival signal for both BVs and LVs. The murine oxygen-induced retinopathy model is a tool that allows for the study of BV regression under hyperoxic conditions. Because the retina is physiologically devoid of LVs, another model must be utilized to study the effects of hyperoxia on LV regression. As our laboratory has demonstrated previously, Prox1-green fluorescent protein (GFP)/Flt1-DsRed transgenic mice can be utilized as a model to observe the physiologic LV regression that occurs postnatally in mice (Zhong et al., 2017). Application of this research tool in conjunction with administration of high concentration oxygen may allow for elucidation of the effects of hyperoxia on LV regression.

In contrast to BVs’ dependence on autocrine VEGF-A, most LVs do not seem to require continuous VEGF-C signaling for homeostasis; intestinal and meningeal lymphatics are cited as exceptions (Stritt et al., 2021). Thus, it is likely that there are more nuances left to be unearthed in lymphatic vessel maintenance signaling. As evidenced by the example of retinopathy of prematurity in the BV context, further characterization of these LV survival signals will allow for an increased understanding of LV regression.

### Inflammation

Inflammation-induced angiogenesis has been studied in a variety of contexts, including herpes simplex virus 1–induced corneal neovascularization, *helicobacter pylori* infection, cancer, and inflammatory bowel disease (Sajib et al., 2018; Filiberti et al., 2020). Sajib et al. (2018) described the intimate relationship between inflammation and angiogenesis, citing the dual role inflammatory markers such as IL-8 and cyclooxygenase-2 may play in both inflammation and promoting angiogenesis. As inflammatory markers may induce angiogenesis, it follows that inhibition of inflammation may block angiogenesis, and indeed, Folkman described this phenomenon as early as Folkman et al. (1983). Summarized by Logie et al. (2010), the literature is rich with examples of glucocorticoids, potent inflammatory inhibitors, preventing inflammation-induced angiogenesis.

**FIGURE 4:**
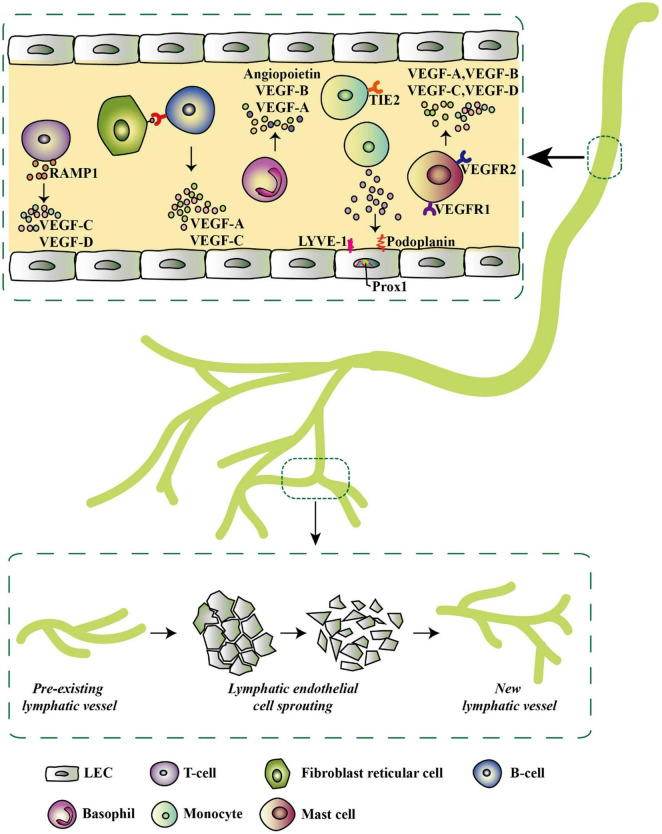
Inflammatory-associated cells and their secretome that initiates lymphatic expansion, reproduced from Ocansey et al. (2021). Most of the inflammatory cells do not only secrete lymphangiogenic factors but also exhibit lymphangiogenic phenotypes by expressing specific lymphatic endothelial markers such as LYVE-1, Prox-1, and podoplanin. These factors trigger pre-existing LVs in the inflammatory environment to give rise to new LVs via LEC sprouting.

Inflammation has also been implicated in lymphangiogenesis (Figure 4; Cursiefen et al., 2006; Kelley et al., 2011; Liao and von der Weid, 2014), and glucocorticoids have been shown to inhibit inflammation-induced lymphangiogenesis (Steele et al., 2011; Martínez-Corral et al., 2012). Although Steele et al. (2011) demonstrated that glucocorticoids inhibit corneal lymphangiogenesis, they found that this anti-inflammatory treatment does not induce LV regression. However, their definition of regressing vessels was determined visually and perhaps further speaks to the lack of a standardized definition for LV regression (Steele et al., 2011). Additionally, this work utilized corneal removal and fixation, therefore necessitating comparison of samples from different mice; a similar methodology using transgenic mice with fluorescent lymphatic detection would allow for continuous live-imaging and determination of the fate of lymphatic vessels, thereby allowing for verification of LV regression. Another study found that upon resolution of inflammation, newly proliferated lymph node LVs do indeed undergo regression (Mumprecht et al., 2012). Further research of the effects of both inflammation resolution and anti-inflammatory therapy on LV regression are warranted and may provide insights into novel mechanisms of LV regression.

### Anti-angiogenic Switch and Negative Feedback

In the context of wound healing, Gosain et al. (2006) described that after robust angiogenesis, exogenous application of pro-angiogenic stimuli, such as VEGF, FGF, and PDGF, is insufficient for preventing the BV regression characteristic of wound healing resolution. Thus, it follows that there must be a summation of anti-angiogenic stimuli that supersede the effects of pro-angiogenic molecules in physiologic BV regression. This concept, termed the anti-angiogenic switch, refers to the negative feedback mechanism by which VECs may be protected from excessive angiogenesis and VEGF stimulation. This notion was referred to as early as 1996 when Lingen et al. (1996) described the ability of retinoic acid to “switch [cultured oral squamous cell carcinomas] from an angiogenic to an anti-angiogenic phenotype”. Over time, proteins in the vasohibin and mammalian Sprouty families have emerged as negative feedback modulators of angiogenesis (Watanabe et al., 2004; Sato and Sonoda, 2007; Wietecha et al., 2011). This research provides evidence for the existence of endogenous mediators that through a negative-feedback mechanism inhibit angiogenesis and prime BVs for physiological regression.

Negative feedback mechanisms are pervasive in biology, as they prevent overstimulation and excessive action. Within the context of inflammation-associated lymphangiogenesis in regional lymph nodes, Kim et al. (2014) described some of the antilymphatic signaling pathways that occur upon inflammation resolution. The authors describe a balancing act between prolymphangiogenic molecules, derived from intranodal follicular B cells, CD11b macrophages, and fibroblast-type reticular stromal cells, and antilymphatic molecules, derived from T lymphocytes. This dynamic tug-of-war accounts for the ability of the lymphatic vasculature to adapt to the situation at hand, with prolymphangiogenic molecules dominating the early phase of inflammation and antilymphatic molecules dominating the resolution of inflammation.

As described by Kim et al. (2014) several antilymphatic molecules derived from T lymphocytes have been identified. Shao and Liu (2006) utilized a pig thoracic duct assay to demonstrate that interferon-α and interferon-γ treatment cause a dose-dependent decline in LEC proliferation *in vitro*; the authors hypothesize this effect may be through the induction of apoptosis (Shao and Liu, 2006). A 2011 study similarly implicated interferon-γ signaling as a main driver in lymph node LV regression (Kataru et al., 2011); through the transfer of T cells to athymic nude mice, demonstrated the role of TH1 cell signaling as a negative feedback regulator of lymph node LV formation. In addition to the effects of TH1 interferon signaling, studies published in 2015 asserted the anti-lymphangiogenic function of TH2 cytokine signaling, with IL-4 and IL-13 as the TH2-derived cytokines implicated in negative regulation of lymphangiogenesis (Savetsky et al., 2015; Shin et al., 2015). Two other studies in 2008 confirmed that TGF-β also serves as a negative regulator of lymphangiogenesis (Clavin et al., 2008; Oka et al., 2008). While the story of negative regulation of lymphangiogenesis is likely incomplete, TH1-derived interferon signaling, TH2-derived IL-4 and IL-13 signaling, and TGF-β signaling all play roles in inhibiting lymphangiogenesis.

### Apoptosis of Regressing Endothelial Cells

The role of apoptosis in regressing BVs seems to be context dependent. Based on high-resolution imaging of murine cornea and zebrafish models, Franco et al. (2015) concluded that BV regression in these models is apoptosis-independent and, instead, reliant upon VEC migration into neighboring vessels. In contrast, the physiological regression of the hyaloid vessels and pupillary membrane occurs in a cell-death dependent manner (Ito and Yoshioka, 1999; Zhu et al., 2000; Kim et al., 2010; Schafer et al., 2020). In consideration of these diametrically opposite means of vascular regression, one involving VEC migration and the other necessitating cell death, apoptosis plays a nuanced role in BV regression. Korn and Augustin (2015) extended the apoptotic mechanism a step further, contending that apoptosis may be the primary driver of BV regression in some cases or a consequence of depleted survival signaling. Through this nuanced understanding of apoptosis in BV regression and much ingenuity, Schafer et al. (2020) were able to repurpose YK-4-279, a small molecule inhibitor of E-26 transformation-specific transcription factors, to further promote apoptosis and enhance hyaloid vessel regression *in vivo*. These findings in a hyaloid vessel regression murine model successfully translated to human *in vitro* culture, demonstrating YK-4-279’s efficacy in regressing human umbilical vein endothelial cell tubes. Their discovery, facilitated by an understanding of apoptosis in BV regression, may prove fruitful in therapeutic applications for pathological neovascularization (Schafer et al., 2020). For an expert review on EC apoptosis in BV regression, readers are referred to the review by Watson et al. (2017).

We have arrived at this nuanced understanding of apoptosis in BV regression as a result of much research, and to our knowledge, there have been few investigations of the role of apoptosis in LV regression. In a study, Hou et al. (2021) utilized ultraviolet A light crosslinking to induce regression of corneal LVs and BVs in an apoptosis-dependent manner. Perhaps in an example of migration-dependent temporary LV regression, Gur-Cohen et al. (2019) demonstrated a transient dissociation of LVs from the hair follicle stem cell niche after the onset of the hair cycle and noted the absence of apoptosis in this context. In Mäkinen et al. (2001) demonstrated that expression of soluble VEGFR-3 in the skin of transgenic mice starting at embryonic day (E)15 lead to regression of dermal LVs with noted apoptosis of LECs in the embryo. Apart from these studies, there has been little research on the role of apoptosis in LV regression. Extending what is known from the BV regression literature, it is similarly possible that LV regression may proceed through migration of LECs into neighboring vessels or through apoptosis of pruned LV segments. Further studies are warranted to unpack the context-specific role of apoptosis in LV regression.

## Pathologies Involving Lymphangiogenic Regression and Dysfunction

In health, the lymphatic system plays a key role in fluid homeostasis, lipophilic nutrient absorption in the gastrointestinal tract, and immune surveillance (Tammela and Alitalo, 2010; Yamakawa et al., 2018; Johnson, 2021). Disruption of these roles can result in interstitial fluid imbalance and edema, nutrient malabsorption, and inflammatory pathologies, respectively (Tammela and Alitalo, 2010; Saito et al., 2013; Abouelkheir et al., 2017). Lymphatic dysfunction has long been implicated in diseases such as lymphedema, tumor metastasis, and chronic inflammatory diseases such as rheumatoid arthritis (Rockson, 2001; Detmar and Hirakawa, 2002; Alitalo, 2011). Although research on the lymphatic system still lags behind that for the vascular system (Adamczyk et al., 2016), dysfunction of lymphatic networks has been increasingly identified as a novel player in different pathologies. For a more complete overview of the recently uncovered roles of general lymphatic dysfunction in pathologies such as obesity, inflammatory bowel disease, glaucoma, cardiovascular disease, and neurodegenerative disease, see the 2020 review by Oliver et al. (2020). Here, we describe pathologies with a recently uncovered LV regression component.

### Neurodegenerative Disease: Lymphatic Vessel Regression in the Aging Brain

Prior to the discovery of meningeal LVs by Louveau et al., 2015, it had long been accepted that the central nervous system lacked a lymphatic vascular system (Louveau et al., 2015; Petrova and Koh, 2020). On the heels of this initial discovery, Antila et al. (2017) utilized *Prox1-eGFP* transgenic mice to modulate and observe meningeal LV development. Through their work, they elucidated that meningeal LVs develop postnatally through VEGF-C and VEGFR-3 signaling. Lymphangiogenesis from meningeal LVs was observed upon injection of AAV-VEGF-C, demonstrating that meningeal LVs are capable of dynamically regressing or undergoing lymphangiogenesis dependent on VEGF-C concentrations. This ability to modulate meningeal LV density may have additional therapeutic implications in neurodegenerative diseases such as Alzheimer’s disease and Parkinson’s disease. In Ma et al. (2017) demonstrated that LVs are the primary conduit for CSF drainage and also observed an age-dependent decline in CSF lymphatic outflow. Considering an age-related decline in CSF outflow along with the important role of CSF in waste removal, Da Mesquita et al. (2018) posited a role for lymphatic flow impairment in Alzheimer’s disease pathology. Their studies of photodynamic meningeal lymphatic ablation in murine models showed that LV impairment translates into impaired efflux of interstitial macromolecules as well as impaired cognitive function (Da Mesquita et al., 2018). Similar studies utilizing mouse models overexpressing α-synuclein demonstrated that LV ligation and the subsequent decrease in CSF clearance increase α-synuclein accumulation, increase neuronal loss, and impair motor function, suggesting that age-related LV dysfunction may also contribute to the pathogenesis of Parkinson’s disease (Zou et al., 2019). In support of this hypothesis, Ding et al. (2021) used magnetic resonance imaging to visualize lymphatic outflow in patients with Parkinson’s disease and found significantly reduced meningeal lymphatic flow in these patients as compared to controls. The current literature suggests that age-related regression of LVs may underpin and exacerbate both Alzheimer’s disease and Parkinson’s disease, and thus, an improved understanding of LV regression could support the development of therapeutics that can modulate this pathological lymphatic impairment.

### Cardiovascular Disease and Atherosclerosis Progression

Lymphatic vessel regression has been hypothesized to exacerbate atherosclerotic cardiovascular disease, the leading cause of death worldwide (Csányi and Singla, 2019; Vourakis et al., 2021). In health, the lymphatic system serves as a conduit for reverse cholesterol transport, the process by which peripheral cholesterol is retrieved and shuttled to the liver by high-density lipoproteins (HDLs) (Huang et al., 2015). Some evidence suggests that reverse cholesterol transport may hamper the pathogenesis of atherosclerosis, although Huang et al. (2015) highlighted that clinical studies have yet to corroborate the athero-protective role of HDL-mediated cholesterol transport (Rosenson et al., 2012). By serving as a channel for cholesterol retrieval, LVs may play a role in reducing the cholesterol burden in atherosclerosis (Fernández-Hernando, 2013; Csányi and Singla, 2019). Studies have suggested that adventitial lymphatics of atherosclerotic vessels undergo regression, leading to potentially impaired drainage of inflammatory cytokines and pro-atherosclerotic lipids, thereby exacerbating atherosclerosis (Taher et al., 2016; Csányi and Singla, 2019). While a component of LV regression may exacerbate atherosclerotic disease, therapeutic lymphangiogenesis is seen to promote recovery of cardiac tissue post-myocardial infarction in mice (Houssari et al., 2020). Using an adeno-associated viral gene delivery of VEGF-C, Houssari et al. (2020) demonstrate that induced-lymphangiogenesis accelerates the resolution of cardiac inflammation following experimental myocardial infarction. A separate work by Liu et al. (2020) demonstrates that LECs play a cardioprotective role through the release of a molecule called Reelin, corroborating the role of lymphatic vessels in cardiac homeostasis. Overall, LVs are hypothesized to have an athero-protective role while LECs are seen to have an important role in injury resolution following myocardial infarction.

## Reflecting on Future Directions

### Models for Studying Lymphatic Vessel Regression

A comprehensive understanding of LV regression will require robust research tools, including appropriate animal models. Zhang et al. (2011), Shi et al. (2020) employed a murine cornea model to observe lymphatic regression. Zhang et al. (2011) proposed that the neovascular privilege of the cornea allows for a wide range of lymphatic growth and plasticity, while Shi et al. (2020) concluded that the cornea has strong potential for studying the effect of aqueous humor on lymphatic vessels (Zhang et al., 2011). The mouse tail has also been identified as a useful tool in lymphatic visualization (Weiler et al., 2019). Researchers have used this model in the context of lymphedema (Bramos et al., 2016; Zhou et al., 2020) and general lymphatic trafficking (Hassanein et al., 2021), but Weiler et al. (2019) were optimistic about its use in many other lymphatic conditions. In the context of Gorham Stout disease, Monroy et al. (2020) studied the extent of lymphatic growth due to pre-existing vessels in mouse bones. Like the cornea, bone is also devoid of lymphatic vessels in a healthy state, and using a model for lymphatic research similar to the cornea could lead to valuable insight into conditions such as Gorham Stout disease. Similarly, Yao et al. (2014) investigated VEGF-C overexpression mice to determine whether this model is useful to study LV development in the respiratory tract but found a condition resembling pulmonary lymphangiectasia, a serious condition in the newborn. Hence, models do not necessarily need to be devoid of LV originally, as this example demonstrates the lung as a valid option to better understand pulmonary lymphatic pathology. A valid model is needed also for future LV regression research in the context of cancer, and Frenkel et al. (2021) proposed the use of long-lived human LECs to better understand lymphangiogenesis and tumor–LV interactions. These cells represent a novel method for studying specifically human tumor lymphangiogenesis and can be further employed to study LV regression. In contrast, sentinel lymph node lymphangiogenesis is a well-documented research model that has been utilized in the study of cancer metastasis (Hirakawa et al., 2005). The lymph node lymphangiogenesis model also has been employed to study LV regression (Mumprecht et al., 2012). Table 3 outlines research models that can facilitate increased understanding of LV regression. Most of these research models are *in vivo* tools that allow for the perturbation and observation of LV dynamics in transgenic mice. The noted LEC microfluidic device represents an emerging area of research with microfluidics; in this example, the microfluidic device enables researchers to exert precise control over interactions between the tumor and LV.

**TABLE 3:** Animal models for the study of LV regression.

Model	References	Utility and findings
Cornea (mouse)	Zhang et al., 2011; Shi et al., 2020	In tandem, these studies demonstrated the utility of the murine cornea for observing *de novo* lymphangiogenesis and LV regression. Due to the ordinarily avascular nature of the cornea, Prox-1 GFP transgenic mice can be utilized for live-imaging of LV progression and regression. The latter reference utilized this technology to demonstrate potentially therapeutic properties of aqueous humor in inducing LV regression.
Tail (mouse)	Bramos et al., 2016; Weiler et al., 2019; Zhou et al., 2020; Hassanein et al., 2021	The mouse tail lymphedema model involves 2-mm deep surgical circumferential excision of the portion of the tail 2 cm distal to the tail base, which disconnects the superficial and deep lymphatics of the tail, thereby locally mimicking lymphedema pathology. Visualization of lymphatic flow involves injection of a fluorescently labeled tracker, such as fluorescein isothiocyanate-dextran, into the mouse tail.
Bone (mouse)	Monroy et al., 2020	This work utilized several transgenic mouse models to visualize *de novo* lymphangiogenesis and LV regression in the context of generalized lymphatic anomaly, a pathology that may be caused by activating mutations of PIK3CA. Prox1-CreERT2;LSL-Pik3caH1047R transgenic mice offer a tamoxifen-inducible system for expression of PIK3CA in LECs. Osx-tTA-TetO-Cre;TetO-Vegfc;mT/mG transgenic mice offer a murine model of Gorham-Stout disease. This model utilizes the bone-specific Osterix promoter to drive a Tet-On system for VEGF-C overexpression in osteoblasts, osteocytes, and chondrocytes. Furthermore, the mT/mG reporter system causes all Cre-positive cells to express GFP. This work highlights the utility of designing transgenic mice to study LV progression and regression in a wide variety of disease contexts.
Lung (mouse)	Yao et al., 2014	CCSP-rtTA; tetO-VEGF-C transgenic mice can be used to study *de novo* lymphangiogenesis in the context of pulmonary lymphangiectasia. This construct allows for doxycycline-induced expression of VEGF-C in Clara cells and alveolar type II cells. These mice were crossed with Prox1-GFP mice to allow for live imaging of LVs, and the triple transgenic mouse allows for an analysis of the effects of VEGF-C to VEGFR-3 signaling in a pulmonary context. Utilization of this model revealed a critical period when VEGF-C expression and resultant lymphangiogenesis produce a pulmonary lymphangiectasia pathology. This model also enables the study of therapeutic modulation of LV regression to treat and prevent pulmonary lymphangiectasia.
Long-lived human LECs	Frenkel et al., 2021	A microfluidic LV model enables analysis of *de novo* lymphangiogenesis and tumor–LV interaction. Lentiviral delivery of human telomerase and BMI-1 expression cassettes was utilized to develop an immortalized human LEC line. This cell line was paired with a microfluidic chip consisting of a free-standing extracellular matrix to visualize the formation of LV-like structures. This model was next co-cultured with mouse colon cancer organoids, enabling live visualization of tumor-induced lymphatic vasculature changes. This microfluidic model can be utilized to mimic both native and tumoral contexts of lymphangiogenesis and LV regression. Application of exogenous therapeutics or molecules of interest to this model can be used to study methods for modulating LV regression and lymphangiogenesis.
Lymph Node	Hirakawa et al., 2005; Mumprecht et al., 2012; Truman et al., 2012	Transgenic mice overexpressing VEGF-A were seen to exhibit sentinel lymph node lymphangiogenesis in a cutaneous squamous cell carcinoma model. While this study by Hirakawa et al., 2005 identified VEGF-A as a tumor lymphangiogenesis inducer, it also demonstrated the utility of the lymph node as a model to study lymphatic vessel dynamics. A different study utilized radiolabeled antibodies against LYVE-1 in conjunction with positron emission tomography to visualize murine lymph node lymphangiogenesis in response to induced inflammation of the skin. Upon resolution of inflammation 3 months later, the authors observed LV regression, thereby demonstrating that inflammation-induced lymph node lymphangiogenesis is indeed reversible. With the progression of transgenic fluorescent reporter mice and robust visualization of LVs in lymph nodes, this remains a powerful tool for exploring lymphatic vessel dynamics.

### Potential Therapeutic Applications of Lymphatic Vessel Regression

In Section “Pathologies Involving Lymphangiogenic Regression and Dysfunction,” we explored pathologies with a component of LV regression. Accordingly, potential avenues exist for the therapeutic use of LV regression, further emphasizing the importance of characterizing LV regression. An understanding of the nuanced mechanisms by which LV regression occurs may facilitate methods to either therapeutically block these mechanisms in cases of pathological LV or therapeutically induce these mechanisms in cases of pathological lymphangiogenesis. Table 4 lists anti-lymphangiogenic agents that have been employed in pre-clinical studies, and Table 5 lists anti-lymphangiogenic modulatory agents that have been tested in clinical trials for their therapeutic potential for a select few pathologies.

**TABLE 4:** Anti-lymphangiogenic agents explored in pre-clinical studies.

Therapeutic agent	References	Context and mechanism of action
Atorvastatin	Ogata et al., 2016	In the context of an early lymphedema murine model, interactions between TH1/TH17 CD4 + T lymphocytes and macrophages result in increased macrophage VEGF-C expression. Daily oral atorvastatin for 1 month reduces the proportion of IFN-γ– and IL-17–secreting CD4 + T lymphocytes, thereby decreasing VEGF-C expression in lesional macrophages. This therapeutic intervention suppresses pathological lymphangiogenesis that exacerbates lymphedema pathology. The authors describe statins as inhibitors of isoprenoids synthesis, thereby resulting in decreased T cell proliferation and differentiation; however, they concede that the exact therapeutic mechanism of atorvastatin in lymphedema is likely still unknown.
Doxycycline	Han et al., 2014	The authors explored the effects of doxycycline on a corneal inflammation-induced lymphangiogenesis murine model. Topical doxycycline application over a 10-day period post corneal injury resulted in dramatically reduced lymphangiogenesis as compared to control mice. Doxycycline was determined to exert its anti-lymphangiogenic effects via overall inhibition of VEGF-C to VEGFR-3 signaling. Additionally, doxycycline application resulted in reduced VEGF-C–induced human dermal LEC proliferation as well as reduction of macrophage-produced lymphangiogenic factors. The authors deduced that the effects of doxycycline were mediated through the PI3k/Akt pathway and by inhibition of matrix metalloproteinases.
TH1 Cytokines	Shao and Liu, 2006; Kataru et al., 2011	Shao and Liu, 2006 utilized a porcine thoracic duct assay to demonstrate the anti-lymphangiogenic effects of interferon-α and interferon-γ *in vitro.* These cytokines, produced by TH1 CD4 + T lymphocytes, were deemed to induce LEC apoptosis. Kataru et al., 2011 demonstrated the anti-lymphangiogenic effects of TH1 signaling on lymph nodes through the transfer of T cells to athymic nude mice. Although interferon-γ signaling was identified as a likely mediator of the anti-lymphangiogenic effects of TH1 cells, the authors noted that other T-cell–derived factors may also exert anti-lymphangiogenic effects. Kataru et al., 2011 identified JAK1-STAT1 signaling as a key pathway in interferon-γ lymphangiogenesis inhibition.
TH2 Cytokines	Savetsky et al., 2015; Shin et al., 2015	TH2 CD4 + T lymphocyte-derived cytokines, namely IL-4 and IL-13, were seen to exert anti-lymphangiogenic effects. IL-4 and IL-13 resulted in the downregulation of essential LEC transcription factors. In an *in vitro* co-culture system of TH2 cells and LECs, TH2 cytokines were seen to inhibit lymphatic tube formation. Shin et al., 2015 demonstrated a therapeutic application of this finding *in vivo* by inhibiting IL-4 and IL-13 in a murine allergic asthma model; neutralizing antibodies against IL-4 and IL-13 resulted in increased lymphatic vessel density, enhanced functioning of lung lymphatic vessels, and subsequent improvement in antigen clearance.
IL-17A	Chen et al., 2010; Park et al., 2018	In the context of TH17-mediated immune responses, Park et al., 2018 described the anti-lymphangiogenic effects of IL-17A, a cytokine secreted by TH17 CD4 + T lymphocytes. The authors utilized a cholera toxin inflammation model and demonstrated that IL-17A suppresses lymphatic markers in LECs as well as inhibits lymphangiogenesis in the resolution phases of inflammation. The authors then utilized an IL-17A–neutralizing antibody to demonstrate increased lymphangiogenesis and lymphatic function. In a separate study pertaining to non-small cell lung cancer however, Chen et al., 2010 found that IL-17 upregulates VEGF-C expression in cancer cells and subsequently increases tumor lymphangiogenesis. The authors noted that the Lewis Lung carcinoma cells utilized express IL-17 receptor; therefore, it is possible that IL-17 may have context-dependent effects on lymphangiogenesis.
TGF- β	Clavin et al., 2008; Oka et al., 2008	In an acute lymphedema model of the murine tail by Clavin et al., 2008, TGF-β1 expression was modulated via the application of a topical collagen gel. Tail wound repair with collagen gel application resulted in decreased TGF-β1 expression, which in turn resulted in accelerated lymphatic vessel formation and improved healing. The authors found TGF-β1 to have dose-dependent effects on decreasing proliferation and tubule formation of LECs. A separate study by Oka et al., 2008 found that TGF-β reduced expression of LEC markers and lymph vessel development even in the presence of the pro-lymphangiogenic ligand VEGF-C. They also found that TGF-β1 plays important roles in negatively regulating lymphangiogenesis.
VEGFR-3 Blockade	He et al., 2002	As VEGF-C/VEGFR-3 signaling is the main driver of lymphangiogenesis, blockade of this interaction has long been utilized to exert anti-lymphangiogenic effects. He et al., 2002 transfected human lung cancer cell lines with a soluble fusion protein VEGFR-3-immunoglobulin and then implanted these tumor cells subcutaneously into severe combined immunodeficient mice. The soluble VEGFR-3 protein expressed by tumor cells inhibited VEGF-C/VEGFR-3 interaction. Through this experiment, the authors determined that inhibition of the VEGF-C/VEGFR-3 interaction can suppress tumor lymphangiogenesis and thereby prevent metastasis to regional lymph nodes; however, metastasis of tumor cells to the lungs still occurred via factors extraneous to VEGF-C signaling.

**TABLE 5:** Lymphangiogenic modulatory agents tested in clinical trials.

Agent name	Reference/identification number	Mechanism of action	Indication	Trial status
IMC-3C5	NCT01288989 Saif et al., 2016	Anti-VEGFR-3 mAb	Colorectal cancer and solid tumors	Phase 1
VGX-100	NCT01288989	Anti-VEGF-C mAb	Advanced solid tumors	Phase 1
Etrasimod	NCT02447302 Nagahashi et al., 2012; Sandborn et al., 2020	Sphingosine 1 phosphate receptor antagonist	IBD	Phase 2
Lymfactin	NCT03658967	Adenovirus gene therapy expressing human VEGF-C	Secondary Lymphedema	Phase 2
Doxycycline	NCT02929121	VEGF-C/VEGFR-3 modulation?	Lymphedema Filariasis	Phase 3
Pazopanib	NCT00827372	VEGFR-1,2,3 inhibitor	Secondary Lymphedema	Phase 2
Ubenimex	NCT02700529	Leukotriene A4 hydrolase inhibitor, biosynthetic enzyme for the anti-lymphangiogenic leukotriene B4	Lymphedema	Phase 2

#### Nuanced Role of Lymphangiogenesis in Lymphedema

Lymphedema is a pathology characterized by deficient lymphatic transport, resulting in excessive regional lymphatic fluid accumulation (Rockson, 2001). Although lymphedema can be a primary disorder, this pathology is more commonly acquired as a secondary disruption of lymphatic vasculature caused by surgery, radiation, infection, or trauma (Rockson, 2001). In addition to disfigurement, chronic lymphedema also poses increased risk for cellulitis and lymphorrhea (Ogata et al., 2016). Transgenic mice models expressing soluble VEGFR-3 acquired a lymphedema-like phenotype due to LV regression and disrupted lymphangiogenesis (Mäkinen et al., 2001). As anti-lymphangiogenic agents are seen to precipitate a lymphedema-like phenotype, the value of therapeutic lymphangiogenesis has been tested in animal models to treat lymphedema (Szuba et al., 2002; Saito et al., 2013). However, Ogata et al. (2016) demonstrated that lymphangiogenesis plays a more nuanced role in the progression of lymphedema; in fact, an initial phase of excessive lymphangiogenesis induced by inflammatory cell and LEC interaction exacerbates the development of lymphedema pathology (Ogata et al., 2016). In a murine lymphedema model, VEGF-C expression was found to be upregulated 4 days after lymphatic obstruction, resulting in the formation of immature LVs; inhibition of this prolymphangiogenic VEGF-C signaling reduced fibrosis and adipogenesis, phenotypic features of lymphedema (Ogata et al., 2016). This increased VEGF-C expression was largely attributed to an interaction between macrophages and CD4 + T lymphocytes. TH1 and TH17 CD4 + T lymphocytes release IFN-γ and IL-17, respectively, resulting in activation of macrophages to upregulate VEGF-C expression. To verify this finding, the authors utilized atorvastatin to reduce the proportion of IFN-γ– and IL-17–secreting CD4 + T lymphocytes. Subsequently, macrophages were seen to secrete less VEGF-C and the phenotype of lymphedema was reduced (Ogata et al., 2016). These studies suggest that an interaction between inflammatory cells in early lymphedema results in lymphangiogenesis that exacerbates the acute lymphedema pathology. Although prior research in animal models has shown a role for therapeutic lymphangiogenesis in lymphedema, this example of aberrant lymphangiogenesis exacerbating early lymphedema pathology calls for caution when designing therapeutics that modulate lymphangiogenic processes (Kim et al., 2014; Ogata et al., 2016).

#### Inflammatory Bowel Disease

Inflammatory bowel disease (IBD), which includes Crohn’s Disease (CD) and ulcerative colitis (UC), is a chronic inflammatory disorder of the gastrointestinal track with an increasing pediatric prevalence worldwide (Ye et al., 2015). Chronic IBD is accompanied by LV proliferation within the lamina propria and submucosa; a human study of UC found that the number of LVs in the lamina propria reflects the severity of disease (Fogt et al., 2004). Rahier et al. (2011) observed an increased density of LVs in both CD and UC, further attributing this process to lymphangiogenesis. Although Kim et al. (2014) reported that aberrant LV function is likely involved in IBD, it remains debated whether increased LV density exacerbates IBD pathology or serves a protective function through inflammation resolution (Kim et al., 2014). To determine if early lymphangiogenesis exacerbates IBD pathology, D’Alessiou et al. (2014) utilized adenoviral induction of VEGF-C in experimental murine models of IBD (D’Alessiou et al., 2014). Their results demonstrated that VEGF-C–induced lymphangiogenesis enhanced lymphatic flow and function, thereby improving intestinal inflammation in IBD. A supporting study utilized a VEGFR-3 blocking antibody in a murine model of IBD, demonstrating that inhibition of VEGF-C/VEGFR-3 signaling results in impaired lymphatic morphology that exacerbates the severity of inflammation (Jurisic et al., 2013). Taken together, these findings may suggest that therapeutic prolymphangiogenic signaling can improve the aberrant lymphatic morphology characteristic of IBD. Although lymphatic flow is essential in limiting the progression of inflammation, further studies are needed to characterize whether the increased LV density early in IBD pathology is pathological or protective (Rahier et al., 2011; Kim et al., 2014).

#### Lymphatic Preconditioning to Reduce Transplant Rejection

**FIGURE 5:**
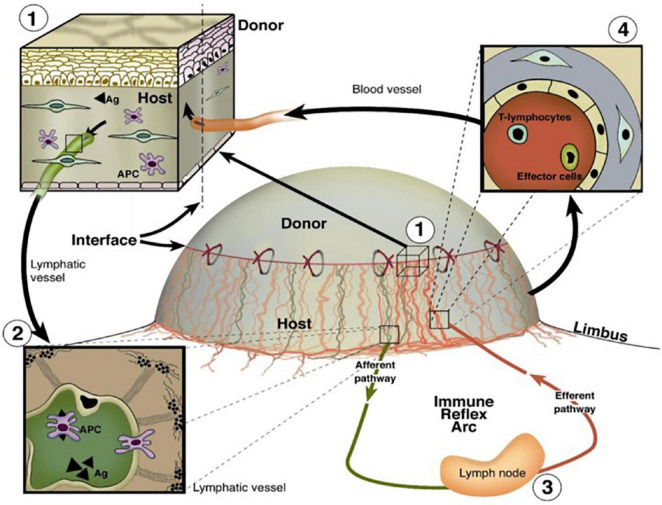
Important roles of BVs and LVs in the high-risk corneal host bed as exit and entry routes of the immune reflex arc leading to immunologic graft rejection, reproduced from Clahsen et al. (2020). (1) Magnification of the host–graft interface where BVs (red) and LVs (green) reach the graft. (2) Antigen (Ag) and APCs both of host and donor can leave the cornea using corneal lymphatics and migrate through corneal LVs to the draining lymph nodes. (3) After stimulation of immune effector cells in the regional lymph nodes, (4) T lymphocytes/effector cells can be released via the efferent blood vessels and gain direct access to the transplant to initiate a rejection reaction (efferent arm of the immune reflex arc).

Despite the advent of human leukocyte antigen matching and the administration of immunosuppressants, allograft rejection remains the leading cause of graft failure beyond 1 year post-transplantation (Yamakawa et al., 2018; Wong, 2020). Lymphangiogenesis has been observed following transplantation of solid organs such as the heart, kidney, and lungs as well as the normally avascular cornea (Wong, 2020; Hou et al., 2021). This proliferation of novel LVs connects the transplanted organ to the systemic lymphatic circulation, thereby facilitating the arrival of antigen-presenting cells (APCs) to draining lymph nodes (Donnan et al., 2021). APC trafficking via LVs to nearby draining lymph nodes can orchestrate the pathological immune response of graft rejection (Donnan et al., 2021). A schematic illustrating the role of the vasculature in immune cell trafficking and corneal graft rejection is shown in Figure 5. Donnan et al. (2021) demonstrated that an increased degree of lymphangiogenesis is positively correlated with the degree of renal transplant rejection, further emphasizing the detrimental role that lymphangiogenesis may play in transplant rejection (Kerjaschki et al., 2004; Phillips et al., 2016; Donnan et al., 2021). Lymphangiogenesis post-transplantation is not always detrimental however, as it may have a protective role following lung transplantation (Wong, 2020).

Due to its immune privileged and avascular nature, the murine cornea also has been utilized as a research model of transplant rejection. Studies have repeatedly demonstrated that corneal neovascularization is correlated with corneal transplantation rejection risk (Bachmann et al., 2010; Le et al., 2018; Hou et al., 2021). Moreover, multiple studies from the Cursiefen group showed that promoting LV regression prior to corneal transplantation in high-risk eyes improves the likelihood of graft survival (Hou et al., 2017, 2018; Le et al., 2018). Hou et al. (2021) described this concept as “lymphangioregressive preconditioning,” as promoting LV regression prior to corneal transplantation in high-risk individuals improved transplantation outcomes. This notion of therapeutic LV regression to prevent graft rejection may also apply in other organ contexts, although further work is required to elucidate the precise roles of LVs in the transplantation of different organs (Hou et al., 2021).

#### Lymphatic Vessel Regression to Prevent Tumor Metastasis

Peritumoral lymphangiogenesis has been increasingly appreciated as a key driver in tumor metastasis and subsequent poor patient prognosis. The first step in metastasis of many cancers involves migration to regional lymph nodes (Detmar and Hirakawa, 2002). As described by Yamakawa et al. (2018), tumor cells can induce lymphangiogenesis from local lymph nodes through pro-lymphangiogenic signaling, effectively creating a highway for lymphatic metastasis. For an expert review on the pre-metastatic niche and tumor signaling in regional lymph nodes, readers are referred to the review by Gillot et al. (2021). Through the secretion of VEGF-C and VEGF-D, tumors of the breast, lung, colon, prostate, and cervix promote the formation of tumor lymphatic networks that serve as routes for metastasis (Detmar and Hirakawa, 2002; Kesler et al., 2013). Multiple studies have confirmed the positive correlation between tumor-induced lymphangiogenesis and tumor metastasis (Wilting et al., 2005; Royston and Jackson, 2009; Nagahashi et al., 2010). Wilting et al. (2005) emphasized that approximately 80% of metastatic tumors coincide with tumor-induced lymphangiogenesis (Wilting et al., 2005).

Given this growing body of evidence that implicates peri-tumoral lymphangiogenesis in metastasis, a major indicator of poor prognosis in cancer, it is evident that therapeutics targeting this process may inhibit the progression of tumors (Khromova et al., 2012). In addition to the blockade of lymphangiogenic signaling, modulating non-overlapping mechanisms of LV regression may serve as another therapeutic avenue to prevent tumor metastasis. The objectives of this review were to summarize novel findings related to LV regression and pose questions for further characterization. Perhaps by inhibiting lymphangiogenesis and also employing novel methods for inducing LV regression, more effective methods for tumor therapy may be developed. Despite this theoretical basis in anti-lymphangiogenic tumor therapy, no anti-lymphangiogenic drugs have been approved for clinical use by the Food and Drug Administration to date (Raica et al., 2016; Okuda et al., 2018; Yamakawa et al., 2018). The exact role of LVs in cancer biology seems to be more nuanced as induced-lymphangiogenesis has also been seen to increase immunosurveillance of a tumor (Vaahtomeri and Alitalo, 2020). Readers are referred herein for a review of the role of LVs in tumor metastasis and immunotherapy (Vaahtomeri and Alitalo, 2020).

## Conclusion

Herein we have highlighted the importance of understanding LV regression in both physiological and pathological contexts and describe potential therapeutic applications for this knowledge. Despite its importance in pathologies such as neurodegenerative diseases, cardiovascular diseases, organ graft rejection, and tumor metastasis, the lymphatic system is largely understudied as compared to its blood vasculature counterpart. By summarizing principles of BV regression and possible extensions to the field of LV regression, we have described areas of study that warrant further exploration. We have summarized research tools that have been utilized to modulate and study LV regression, so that future work can build upon this foundation. Although the lymphatic system and its roles in diverse pathologies have yet to be fully characterized, this review aims to accelerate the work required to better characterize LV regression so that this phenomenon may be modulated for therapeutic purposes.

## Author Contributions

FM, RB, MR, AK, J-HC, and DA contributed to the writing of the manuscript.

## Conflict of Interest

The authors declare that the research was conducted in the absence of any commercial or financial relationships that could be construed as a potential conflict of interest.

## Publisher’s Note

All claims expressed in this article are solely those of the authors and do not necessarily represent those of their affiliated organizations, or those of the publisher, the editors and the reviewers. Any product that may be evaluated in this article, or claim that may be made by its manufacturer, is not guaranteed or endorsed by the publisher.

## Glossary

AAV-VEGF-C, adeno-associated virus encoding VEGF-C; a-MSH, alpha-melanocyte-stimulating hormone; ANG2, angiopoietin-2; APCs, antigen-presenting cells; bFGF, basic fibroblast growth factor; BMI-1, B lymphoma Mo-MLV insertion region 1 homolog; BV, blood vessel; CCL21, chemokine (C-C motif) ligand 21; CCSP-rtTA; Clara cell secretory protein reverse tetracycline-controlled transactivator; CD31, cluster of differentiation 31; Celsr1, cadherin EGF LAG seven-pass G-type receptor 1; CRIM1, cysteine-rich transmembrane BMP regulator 1; CSF, cerebrospinal fluid; CXCL10, C-X-C motif chemokine ligand 10; CXCR3, C-X-C motif chemokine receptor 3; DLL4, Delta-like canonical Notch ligand 4; E15, embryonic day 15; ETS, transcription factor E26 transformation–specific sequence; Fas, Fas cell surface death receptor; Fat4, FAT tumor suppressor homolog 4; FGF2, fibroblast growth factor 2; Foxc2, Forkhead box C2; Gata2, transcription factor binds to the DNA sequence “GATA”; HDL, high-density lipoprotein; HEY, Hairy/enhancer-of-split related with YRPW motif protein; HIF-1, hypoxia-inducible factor 1; IA, intussusceptive angiogenesis; IL-13, interleukin 13; LEC, lymphatic endothelial cell; LV, lymphatic vessel; LYVE-1, lymphatic vessel endothelial hyaluronan receptor 1; Nrp1, neuropilin 1; PDGF, platelet-derived growth factor; PDGFR-α/β,platelet-derived growth factor receptor; PIK3CA, phosphatidylinositol-4,5-bisphosphate 3-kinase catalytic subunit alpha; Pkd1, polycystic kidney disease 1; Prox-1, prospero-related homeobox 1; ROP, retinopathy of prematurity; SA, sprouting angiogenesis; Sema3a, semaphoring 3a; TGF-β,transforming growth factor beta; TIE, tyrosine kinase with immunoglobulin-like and EGF-like domains; TLOs, tertiary lymphatic organs; TSP-1, thrombospondin-1; Vangl2, VANGL planar cell polarity protein2; VEC, vascular endothelial cell; VEGF-A, vascular endothelial growth factor A; VEGF-B, vascular endothelial growth factor B; VEGFR-1/2, vascular endothelial growth factor receptor 1/2; VEGFR-3, vascular endothelial growth factor receptor 3; VIP, vasoactive intestinal peptide; vWF, von Willebrand factor.
